# Immunohistochemical Expression of BRCA1 Protein, ER, PR and Her2/neu in Breast Cancer: A Clinicopathological Study

**DOI:** 10.31557/APJCP.2020.21.4.1025

**Published:** 2020-04

**Authors:** Israa A Hussein, Shatha Th Ahmed, Ameer D Hameedi, Rana Z Naji, Layth Alharbawi, Muzahm Alkhaytt, Intisar S Pity

**Affiliations:** 1 *MBChB, FIBMS Pathology, Department of Pathology, College of Medicine, University of Baghdad, *; 2 *MBChB, PhD mol. Pathology/UK, *; 4 *MBChB, CABS, General Surgeon, College of Medicine, *; 5 *MBChB, FRCS, President of Nineveh University, Ninevah University, *; 3 *MBChB, FIBMS Pathology,Oncology Teaching Hospital, *; 6 *MD, FIBMS, Pathology, College of Medicine, University of Duhok, Baghdad, Iraq. *

**Keywords:** Breast cancer, BRCA oncoprotein, immunohistochemistry

## Abstract

Breast cancer is a heterogeneous hormone-dependent disease. Potential prognosis depends on the clinicopathological evaluation and assessment of other prognostic indicators. The detection of the oestrogen Receptor (ER), Progesterone Receptor (PR), Human epidermal growth factor receptor 2 (Her2/neu) and BRCA1 oncoprotein is pivotal for prognostic evaluation and to choose the appropriate post-surgical adjuvant therapy beside selecting the proper candidate for genetic counselling.

**Objectives:**

To detect the immunoexpression of the BRCA1 oncoprotein in mammary invasive ducal carcinoma and its association with the prognostic markers (ER, PR and Her2/neu hormonal receptors) and other clinicopathological parameters to improve the patients’ treatment plans.

**Methods:**

A cross-sectional study design including 83 paraffin blocks and histological slides collected from Al-Jumhoori Medical City Teaching Hospital Laboratory in Mosul and the Central Public Health Laboratory in Baghdad between the 1st of January 2010 to the 13th of March 2012 for patients diagnosed with primary invasive ductal breast carcinomas. Immunohistochemistry (IHC) using monoclonal antibodies against ER, PR, Her2/neu receptors and BRCA1 protein was performed via the fully automated immunostaining instrument ‘Ventana Benchmark’.

**Results:**

BRCA1 protein immunoexpression was detected in 20.5% of cases. It was significantly high with increasing tumour grade and stage. Although there was a trend of BRCA1 negativity toward negative ER, PR and Her2 receptors, no significant associations were observed with any of these parameters and the patients’ age.

**Conclusion:**

Altered BRCA1 expression is significantly associated with advanced tumour grade and stage. High number of cases with negative BRCA1 expression showed negative ER, PR and Her2/neu expression.

## Introduction

Breast cancer (BC) is one of the most common cancers worldwide and is the second leading cause of death among females in the United States (NBCF, 2018). In Iraq, it constitutes around one-third of the overall registered female tumours (Alwan, 2014). Although the backbone for the treatment of BC is surgical resection, the dilemma for convenient post-operative adjuvant therapies is still prevalent. In order to decide adjuvant therapies to improve the patients’ prognosis and survival rates, a thorough assessment of the clinicopathological state, hormonal testing for (oestrogen, progesterone and Her2/neu) receptors in addition to genetic receptors like BRCA1 and BRCA2 are needed. This can be attributed to the fact that 80% of women with familial tendency to breast and/or ovarian cancer harbour these highly penetrating mutated genes (Shulman, 2010).

The acronym (BRCA) type 1 or 2 stands for breast cancer susceptibility type 1 (BRCA1) and type2 (BRCA2) which are considered as tumour suppressor genes mapped on chromosome 17q21 and 13q12 respectively (Ripperger et al., 2009). These genes can help in repairing DNA damages and prevent uncontrollable cell growth with the inhibition of ER transcriptional activity in the human mammary tissues (Verma et al., 2018; Archey and Arrick, 2017; Wang and Di, 2014). Studies demonstrated that premenopausal women inheriting BRCA1 or BRCA2 gene might be at a higher risk (4 out of 5) for developing breast and/or ovarian cancer as compared to other females of the same age group (1 out of 8) (NBCF 2018; Antoniou et al., 2003; Malone et al., 2000). To the best of our knowledge, limited studies were performed on BRCA immunoexpression and hormone receptor status in our locality, henceforth, this research was performed to check BRCA1 immunoexpression and its association with ER, PR and Her2/neu immunoexpression in infiltrative ductal carcinoma of the breast. 

## Materials and Methods

An observational cross-sectional study involving 83 paraffin blocks and slides for female patients proven histologically as infiltrative duct carcinoma (not otherwise specified) were collected from Al-Jumhoori Medical Teaching Hospital laboratory in Mosul and Medical City Teaching Hospital in Baghdad, Iraq for the period from the 1st of January 2010 to the 13th of March 2012. Patient’s data with regards to their age, histological type, pathological stage and lymph node involvement were retrieved from their medical reports. The tumour histological grading was decided upon examining the H&E stained slides microscopically using ‘Bloom Richardson’s grading system’ (tubular formation, nuclear pleomorphism, hyperchromatism and number of mitosis). The tumour stage was reported after examining all microscopic slides of the excised tumour, adjacent tissues and the dissected lymph nodes in addition to the clinic-radiologic data when available. Four µm thick sections of the paraffin-embedded blocks were obtained using the fully automated immunostaining instrument ‘VentanaBenchmark’ (Dako Denmark, Link 48) for immunohistochemistry (IHC) staining using monoclonal antibodies against ER, PR, Her2/neu receptors (ready to use biogenex kit) as described previously by (Ahmed et al., 2018).

**Figure 1:**
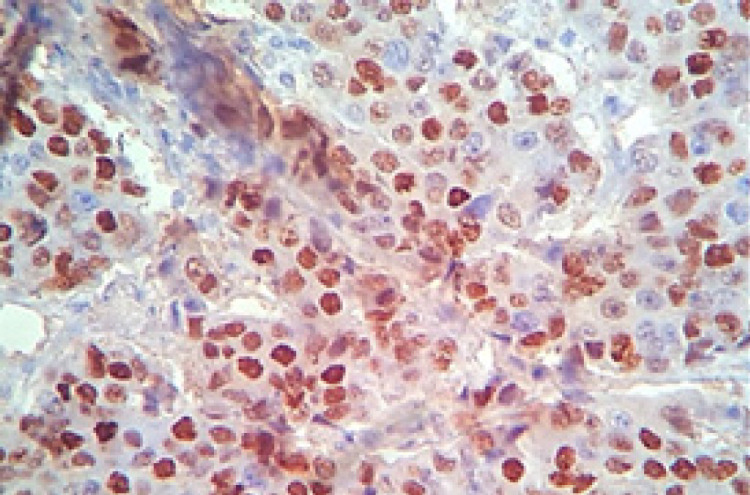
Breast Infiltrative Duct Carcinoma with Positive Nuclear Staining for ER (IHC. X400)

**Figure 2:**
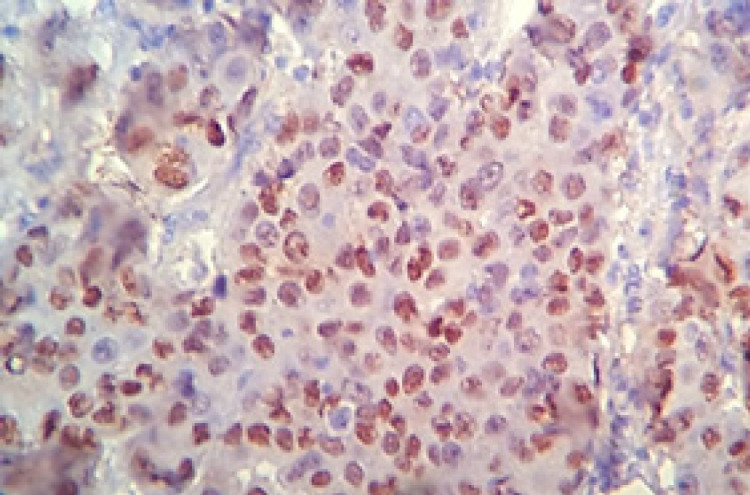
Breast Infiltrative Duct Carcinoma with Positive Nuclear Staining for PR (IHC. X400).

**Figure 3:**
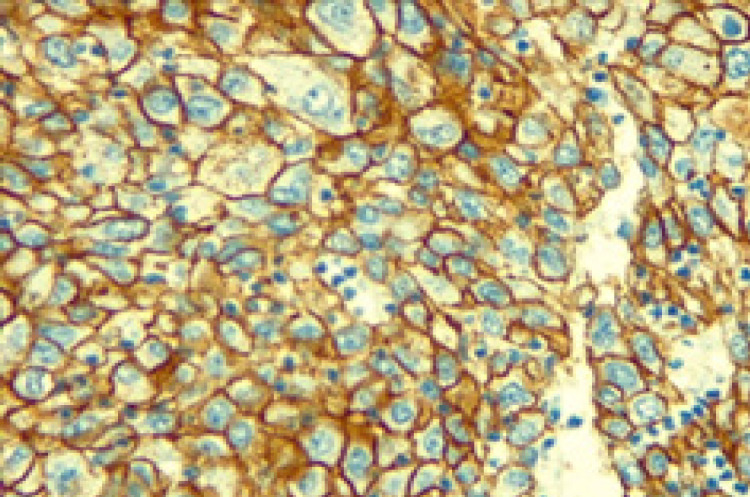
Breast Infiltrative Duct Carcinoma with Positive Membraneous Staining for Her2/neu (IHC. X400).

**Figure 4:**
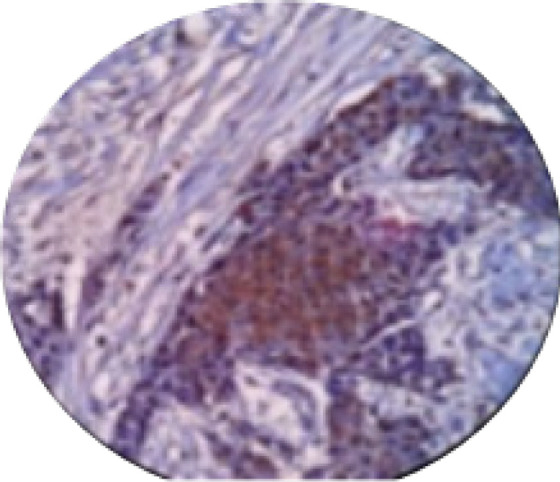
Breast Infiltrative Duct Carcinoma with a Diffuse Nuclear Staining of Weak to Moderate Intensity for BRCA1(IHC. X100)

**Figure 5:**
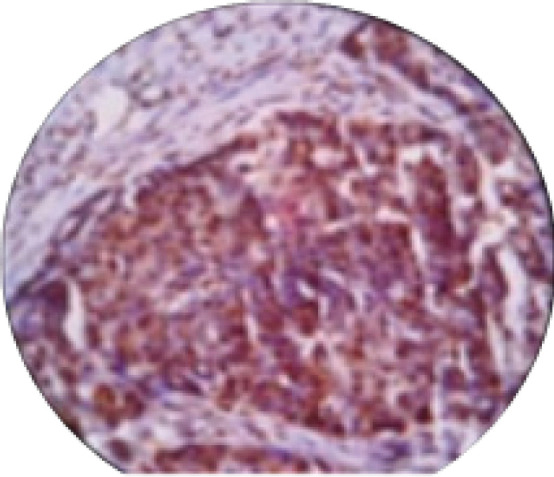
Breast Infiltrative Duct Carcinoma with a Diffuse Nuclear Staining of Moderate Intensity for BRCA1(IHC. X100)

**Figure 6:**
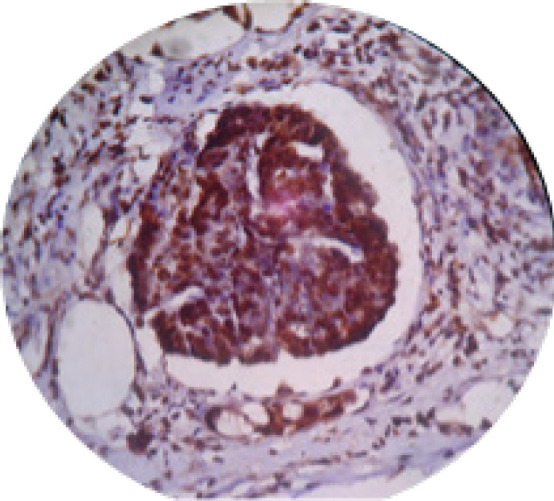
Vascular Invasion with Breast Carcinoma with Nuclear Positivity for BRCA1(IHC,X100).

Oestrogen and progesterone receptors were considered positive when ≥10% of cell nuclei were positively stained (Figures 1 and 2). These two markers were tested only on 63 cases. For Her2/neu testing was limited to 32 cases owing to lack of the kit at the time of the study. Only complete circumferential membranous staining in >10% of tumour cells (3+) was considered positive (Figure 3). For BRCA1 immunostaining, Mouse monoclonal anti BRCA1’ clone MS110 (USA) was used at a dilution of 1:10, then scored as negative (greatly reduced or absent) if the brownish nuclear stain was <20% while nuclear staining >20% was considered as positive (Figures 4-6) (American society of clinical oncology/college of American pathologist clinical practice guideline update 2013).

## Results

Patient’s ages (n= 83) ranged between 27 and 75 years (mean= 47.1 years), forty-nine females (59%) were premenopausal (< 50 years of age). Among the 83 participants, BRCA1 oncoprotein expression was negative (reduced or absent) in 66 (79.5 %) and positive in the remaining 17 cases (20.5%). Although the peak age of BRCA1 negative patients was in the fourth decade, no significant correlation found between BRCA1 expression and different age groups (Table 1). On the other hand, 47 (56.6%) of the tumors were in stage III, 34 (41%) stage II and only two cases were in stage I. Tumor grade 3 was the most frequent 41 (49.4%) cases versus 29 (34.9%) and 13 (15.6%) patients with grade 2 and grade 1 respectively. A statistically significant correlation was found between BRCA1 expression with the stage and grade of the tumor (p-value<0.05) (Table 2). Out of 63 cases tested for ER and PR, 21 (33.33%) were ER positive and 25 (39.6%) were PR positive. Out of the 32 Her2/neu tested cases, 20 (62.5%) were Her2/neu -ve. Fourteen (63.63%) of BRCA1- cases were Her2/neu -ve versus 8 (36.36%) cases with Her2/neu +ve. For BRCA+ve cases, 6 out of 10 cases (60%) were negative for Her2/neu and the remaining 4 cases were Her2/neu +ve. There were no significant correlations between BRCA1 expression and Her2/neu status (Table 3).

Correlating ER/PR subgroups with BRCA1 expression displayed that Phenotype ER-PR- reported in 33 case (52.38%) forming the highest incidence, followed by ER+PR+ 17 (26.98%) and ER-PR+ 9(14.28%). Phenotype ER+PR- 4(6.34%) registered the lowest incidence. Analysis of the correlation of these subgroups with BRCA1 showed that 29 (52.72%) of the BRCA1-ve cases were ER-PR-, followed by ER+PR + /BRCA1-ve 15(27.27%). ER-PR+/BRCA1-ve 7(12.72%) and the least one was ER+PR-BRCA1-ve 4 (7.27%) (Table 4). Although the highest number of BRCA1 cases were ER-PR-, there were no significant correlations between BRCA1and ER, PR status (p-value>0.05).

**Table 1:** Correlations between BRCA1 Expression with Different Age Groups of the Study Population

Age group (years)	BRCA1^1^ (+) No. (%)	BRCA1 (-) No. (%)	Total No. (%)	*p-value*
20-29	0 (0%)	4 (6.06%)	4 (4.81%)	>0.05
30-39	4 (23.5%)	5 (7.57%)	9 (10.84%)	
40-49	4 (23.5%)	32 (48.48%)	36 (43.37%)	
50-59	6 (35.29%)	23 (34.84%)	29 (34.9%)	
60-69	1 (5.88%)	2 (3.03%)	3 (3.61%)	
>70	2 (11.76%)	0 (0%)	2 (2.40%)	
Total	17 (100%)	66 (100%)	83 (100%)	

^1^, BRCA1 denotes to breast cancer susceptibility type 1.

**Table 2:** Correlations between BRCA1 Expression with Tumour Stage and Grade

	BRCA1^1^ (+) No. (%)	BRCA1 (-) No. (%)	Total No. (%)	*p-value*
Stage				
I	0 (0.0)	2 (3.0)	2 (2.4)	<0.05*
II	3 (17.6)	31 (47.0)	34 (41.0)	
III	14 (82.4)	33 (50.0)	47 (56.6)	
IV	0 (0.0)	0 (0.0)	0 (0.0)	
Total	17 (100.0)	66 (100.0)	83 (100.0)	
Grade				
I	4 (23.5)	9 (13.6)	13 (15.6)	< 0.05*
II	1 (5.8)	27 (40.9)	29 (34.9)	
III	12 (70.5)	30 (45.45)	41 (49.4)	
IV	0 (0.0)	0 (0.0)	0 (0.0)	
Total	17 (100.0)	66 (100.0)	83 (100.0)	

^1^, RCA1 denotes to breast cancer susceptibility type 1.

**Table 3:** Frequency and Percentage of ER, PR, Her2/neu Expression in BRCA1(+ve) and BRCA1(-ve) Cases

	BRCA1^1^ (+) No. (%)	BRCA1 (-) No. (%)	Total No. (%)	*p-* *value*
Oestrogen Receptors (ERs)				
ER^2^ +	2 (28.5)	19 (33.9)	21 (33.33)	>0.05
ER -	5 (71.4)	37 (66.07)	42 (66.66)	
Total	7 (100.0)	56 (100.0)	63 (100.0)	
Progesterone Receptors (PRs)				
PR^3^ +	3 (42.85)	23 (41.07)	25 (39.6)	>0.05
PR -	4 (57.15)	33 (58.93)	38 (60.4)	
Total	7 (100.0)	56 (100.0)	63 (100.0)	
Her2/neu Receptors				
Her2/neu^4^ +	4 (40)	8 (36.36)	12 (37.5)	>0.05
Her2/neu -	6 (60)	14 (63.63)	20 (62.5)	
Total	10 (100.0)	22 (100.0)	32 (100.0)	

^1^, BRCA1 denotes to breast cancer susceptibility type 1; ^2^, ER denotes to oestrogen receptors; ^3^, PR denotes to progesterone receptors; ^4^, Her2/neu denotes to human epidermal growth factor receptor 2.

**Table 4.** Correlation between ER, PR Subtypes Expression in BRCA1(+) and BRCA1(-) Cases

ER, PR subgroups	BRCA1 (+) No. (%)	BRCA1 (-) No. (%)	Total No. (%)	*P-value*
ER+PR+	2 (28.57)	15 (27.27)	17 (26.98)	>0.05
ER-PR-	4 (57.14)	29 (52.72)	33 (52.38)	
ER+PR-	0 (00%)	4 (7.27)	4 (6.34)	
ER-PR+	1 (14.2)	7 (12.72)	9 (14.28)	
Total	7 (100.0)	55 (100.0)	63 (100.0)	

## Discussion

In the current study using immunohistochemistry, 20.4% were positively stained for BRCA1 whereas Verma et al. reported 55.6% nuclear positivity for BRCA1 in their study (Verma et al., 2018). A significant relation between BRCA1 expression and advanced tumour stage was found which coincides with the reports from other researches (Bugrein and Bujassoum, 2016; Ashraf et al., 2011). Additionally, we found that there is a significant association between BRCA1 immunoexpression and graded tumours, mostly grade III (49.4%). This came in contrast to Yang et al. who demonstrated a significant inverse relationship between BRCA1 expression and histologic grade (Yang et al., 2001). These findings regarding the advanced stage and high grade with BRCA1 expression may reflect the poor prognosis of tumours harbouring this gene mutation (Hedau et al., 2015; Yang et al., 2001).

An interesting finding in the current study was the high frequency of negative ER and PR were observed in BRCA1 negative group. Despite not being statistically significant, a trend of Her2/neu negative BCs toward lack of BRCA1 expression was observed. Ansquer et al. argued that BRCA1 expression was not related to Her2/neu expression (Ansquer et al., 2005). A considerable number of studies demonstrated negative ER, PR and Her2/neu in familial BC (Yang et al., 2015; Chappuis et al., 2000; Henderson and Patek, 1998). Similar findings have been reported in other literatures (Verma et al., 2018; Kumar et al., 2017; Ashraf et al., 2011).

In the same line, Jóhannsson et al., (1997) observed a significant lower frequency of ER/PR positive expression in BRCA1 tumours using immunohistochemical staining of paraffin sections. However, higher values were reported by others (Geredeli et al., 2019; Huszno et al., 2019; Verma et al., 2018). Such differences could be attributed to the variations in the intra and inter laboratory technical immunostaining methods (Iqbal and Buch, 2016). Other studies reported that the majority of BRCA1-related tumors were grade III invasive ductal cancers and were ER negative regardless of the age and stage of the disease (Krammer et al., 2017). This raises the concept that ER negativity is neither a sequel of younger ages nor the graded tumours at onset of diagnosis, but is an intrinsic character related to BRCA1 positive cancers (Shuen and Foulkes, 2012; Foulkes et al., 2004). Similarly, Tung et al, reported in 2010 that Patients younger than 50 years diagnosed with BC were significantly more likely to have an ER negative cancer compared with older aged patients (Tung et al., 2010). It is important to mention that considerable percentages of negative ER may be “false negative” due to some technical factors related to inappropriate tissue fixation, antigen retrieval or staining a necrotic areas (Mouttet et al., 2016; Nadji et al., 2005). Moreover, the lack of standardised measurements of grade and ER increases the errors in the results (Foulkes et al., 2004). Furthermore, the cut off point for negativity of ER/PR in most studies is calculated as immunostained active cells<10% whereas the new guidelines consider negative ER/PR as <1% (Mouttet et al., 2016).

In conclusion, our study suggests that BRCA1 expression is significantly correlated with an advanced tumour grade and stage. A higher number of cases with negative BRCA1 immunoexpression showed negative ER, PR and Her2/neu expression. These findings raise the importance of identifying molecular profiling of ER, PR, Her2/neu and the state of BRCA1 expression as useful prognostic markers for BC patients before starting treatment and selecting proper persons for the genetic screening of the mutated BRCA1 oncoprotein.
